# Chronotropic incompetence in end-stage liver disease

**DOI:** 10.1371/journal.pone.0270784

**Published:** 2022-08-01

**Authors:** Renata Główczyńska, Sonia Borodzicz-Jażdżyk, Michał Peller, Joanna Raszeja-Wyszomirska, Piotr Milkiewicz, Krzysztof Zieniewicz, Grzegorz Opolski

**Affiliations:** 1 1^st^ Department of Cardiology, Medical University of Warsaw, Warsaw, Poland; 2 Chair and Department of Experimental and Clinical Physiology, Laboratory of Centre for Preclinical Research, Medical University of Warsaw, Warsaw, Poland; 3 Liver and Internal Medicine Unit, Department of General, Transplant and Liver Surgery, Medical University of Warsaw, Warsaw, Poland; 4 Department of General, Transplant and Liver Surgery, Medical University of Warsaw, Warsaw, Poland; Cincinnati Children’s Hospital Medical Center, UNITED STATES

## Abstract

**Background:**

Cirrhosis causes alterations in the cardiovascular and autonomic nervous systems and leads to cirrhotic cardiomyopathy (CCM). CCM is defined as cardiac dysfunction characterized by an impaired systolic responsiveness to stress or exercise, and/or impaired diastolic function, as well as electrophysiological abnormalities, including chronotropic incompetence (CI), in the absence of other known cardiac disease. CI is a common feature of autonomic neuropathy in cirrhosis. The aim of the study is to assess the role of cardiac exercise stress test in the diagnosis of CCM.

**Methods:**

The analysis included 160 end-stage liver disease (ESLD) patients who underwent a cardiac exercise stress test prior to the orthotopic liver transplantation. CI was defined as the inability to achieve the heart rate reserve (HRR). Pertaining to the therapy with beta-blockers: 80% of HRR was achieved in patients not taking beta-blockers and 62% in patients taking beta-blockers.

**Results:**

In the analyzed population, 68.8% of patients met the criteria for CI. CI was more frequent in the more severe ESLD (with a higher MELD score and in a higher Child-Pugh class). In comparison to the viral hepatitis and other etiologies of ESLD, patients with alcoholic cirrhosis had a significantly lower rest heart rate (HR), lower maximal HR, lower median achieved percentage of maximal predicted HR (MPHR), a smaller percentage of patients achieved ≥ 85% of MPHR and a lower heart rate reserve. No significant relationship between the survival of OLT recipients and presence of chronotropic incompetence regarding to class of Child-Pugh scale, MELD score and etiology of ESLD were found.

**Conclusions:**

The prevalence of CI is higher among liver transplant candidates than previously described. The altered chronotropic response may differ in regard to the severity of liver disease correlating with both the Child-Pugh and MELD scores, however CI does not seem to influence the long-term survival post OLT. Exercise stress test is a reliable, safe and useful tool for the diagnosis of CCM in liver transplant candidates and should be included in the standard cardiovascular assessment prior to OLT.

## 1. Introduction

Cirrhosis leads to cardiac and circulatory dysfunctions due to splanchnic arterial vasodilation. Initially, deterioration of the hemodynamic function is compensated by the development of a hyperkinetic circulation. Furthermore, the progression of liver disease and portal hypertension leads to progressive vasodilatation, which reduces the effective arterial blood volume and results in the activation of the sympathetic nervous system as well as the renin-angiotensin-aldosterone system (RAAS). These circulatory disturbances can lead to the dilatation of the left cardiac chambers and the development of functional changes in the heart [1].

In 2005 the World Gastroenterology Organization proposed a working definition of cirrhotic cardiomyopathy (CCM), which is defined as a cardiac dysfunction with impaired contractile responsiveness to stress and/or altered diastolic relaxation with electrophysiological abnormalities in the absence of other known cardiac disease [2]. The abovementioned electrophysiological disorders include prolonged QT interval, electromechanical uncoupling and chronotropic incompetence (CI) [2]. CI, a common feature of the autonomic neuropathy in cirrhosis, is defined as the inability to efficiently increase heart rate (HR) or myocardial contractility in response to physical exercise or pharmacological stimulation [3, 4]. It should be emphasized that the definition of CCM is only a working one and requires further clarification. In addition, there has been no clear definition or criteria created for the diagnosis of CI yet, which makes it difficult to identify patients with CCM.

End-stage liver disease (ESLD), regardless of the etiology, is a leading indication for orthotopic liver transplantation (OLT) [3, 5]. In the last two decades, significant improvements in the preoperative work-up, surgical technique and intraoperative care of OLT recipients as well as the tailored immunosuppressive therapy have resulted in decreased perioperative complications and increased patient survival. However, cardiovascular complications are still a major cause of morbidity and mortality after OLT. Thus, limited cardiac reserve preoperatively may be associated with poor outcomes postoperatively, including the perioperative hemodynamic instability [5]. The goal of the cardiovascular evaluation in OLT candidates is the assessment of intra and post-operative survival prognosis and detection of severe cardiopulmonary disease, which makes OLT futile. Several studies formulated the optimal risk stratification in patients undergoing OLT. However, a considerable controversy surrounding the screening methodology still persists. It is crucial to identify patients with CCM, who may potentially have a high risk of perioperative and postoperative mortality. Therefore, the aim of the study is to assess the role of cardiac exercise stress test in the diagnosis of CCM.

## 2. Methods

### 2.1. Study population

**Fig 1:**
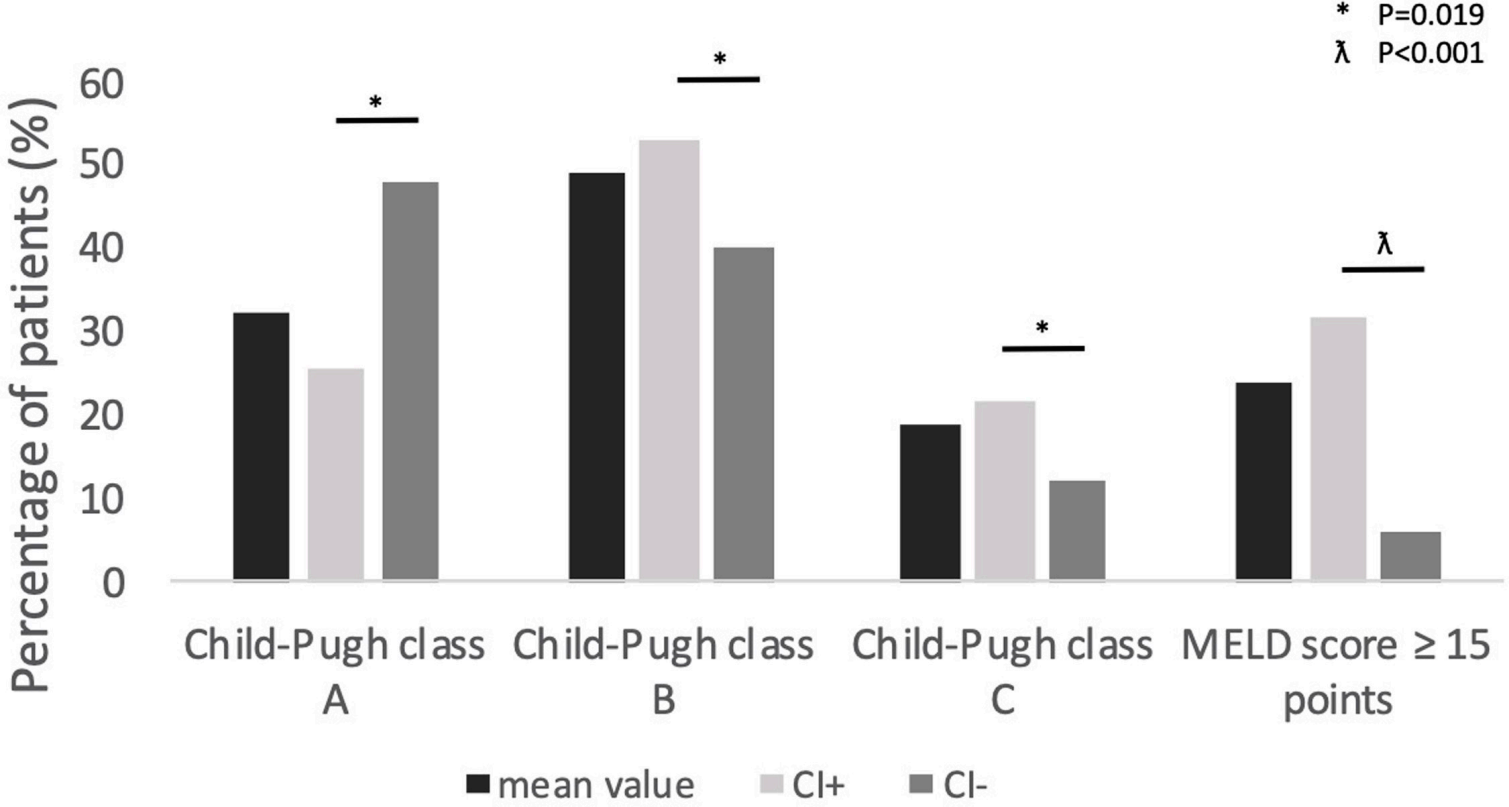
Severity of end stage liver disease in the analyzed group of patients. MELD—Model for End-Stage Liver Disease.

This single-center retrospective analysis included 160 patients (61.9% males) diagnosed with ESLD, who were consulted by a designated cardiologist in our center and who underwent the OLT (Table 1 and Fig 1). As part of the cardiac evaluation before OLT, the cardiac exercise stress test was performed in all patients enrolled in the study. It should be emphasized that the exercise stress test was used only as a functional assessment of patients prior to OLT and its poor result itself did not disqualify patients from the OLT. The mean time from the cardiac exercise stress test to the OLT was 110 ± 100 days. This study used a register-based follow-up design. We used the Polish Civil Personal Registration Number, which is unique to each Polish citizen, to link information from the national registry of deaths at the Ministry of the Interior and Administration to the clinical data obtained in this study. Observations were completed at the date of last available follow-up. The mean follow-up period was over 2 years (736.5 ± 260.6 days). The study protocol was approved by the Bioethics Committee at the Medical University of Warsaw. The patients signed an informed consent to have data from their medical records used in research.

**Table 1:** Baseline characteristics of patients.

Characteristic/Variable	Value	CI (+)	CI (-)	p
Demographics				
Age, years (mean ± SD)	49 ± 12.4	48 ± 13	52 ± 10.4	0.173
Gender, male (n; %)	99; 61.9%	63.3%	58.0%	0.599
Anthropometrics				
Height (cm)	169.4 ± 9.5	170.2 ± 9.7	167.8 ± 8.8	0.478
Weight (kg)	72.9 ± 14.4	71.9 ± 14.9	75.0 ± 12.9	0.19
BMI (kg/m2)	25.3 ± 4.1	24.7 ± 3.8	26.7 ± 4.4	0.005
Baseline cardiovascular parameters				
Resting HR (b.p.m.)	84.3 ± 18.5	81.5 ± 18.5	90.5 ± 16.9	0.005
SBP (mmHg)	111.0 ± 14.2	110.6±14.5	111.9 ± 13.5	0.612
DBP (mmHg)	76.0 ± 7.5	75.8 ± 7.4	76.6 ± 7.7	0.548
Medications prior to exercise test				
Beta-blockers (n; %)	73; 45.3%	45.1%	46.0%	1.00
Etiology of liver disease				
Alcoholic (n; %)	29; 18.1%	21.8%	10.0%	0.179
Viral hepatitis (Hepatitis B or C) (n; %)	72; 45%	41.8%	52.0%	
Other (n; %)	59; 36.9%	36.4%	38.0%	
Complications of ESLD				
HCC (n; %)	32; 20%	16.4%	28.0%	0.094
Ascites (n; %)	43; 26.9%	29.1%	22.0%	0.442
Gastroesophageal varices grade III-IV (n; %)	50; 31.3%	32.7%	28.0%	0.586
History of bleeding from gastroesophageal varices (n; %)	29; 18.1%	19.1%	16.0%	0.825
History of overt encephalopathy (n; %)	31; 19.4%	23.2%	12.0%	0.132

CI–chronotropic incompetence, ESLD–End stage liver disease, HCC–hepatocellular carcinoma

### 2.2. Exercise stress test

The exercise stress test was performed according to the Bruce protocol where the speed and gradient of the treadmill were raised every 3 minutes. A standard 12-lead electrocardiogram was performed before, during and after the stress test until the recovery of the heart rate. Blood pressure was measured before, every 3 minutes during the exercise test and after the recovery. The exercise tests were terminated due to the fatigue, chest pain, dyspnea, achievement of maximal predicted heart rate (MPHR) or on patient demand. Beta-blockers were not withdrawn prior to the exercise test.

### 2.3. Laboratory data

Blood samples were collected on the same day as the exercise stress test, for the calculation of the Model for End-Stage Liver Disease (MELD) score:


MELDscore:9.57(Lnserumcreatinine)+3.78(Lnserumbilirubin)+11.20(Lninternationalnormalizedratio)+6.43

### 2.4. Chronotropic incompetence

**Fig 2:**
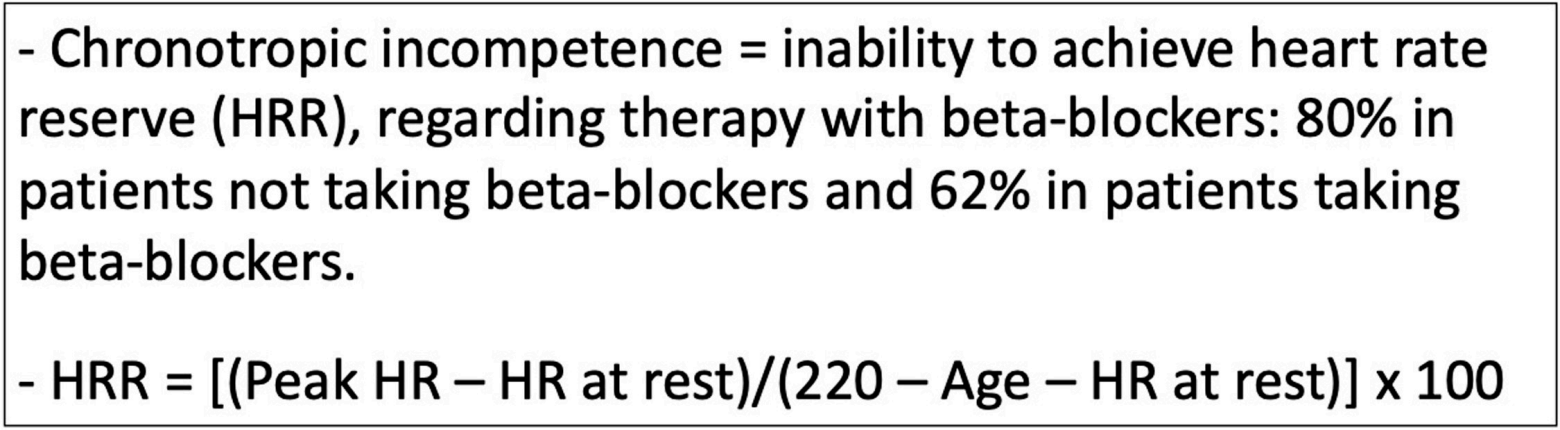
Definition of chronotropic incompetence.

The exercise stress test was diagnostic if the patient was able to achieve 85% of MPHR, which was calculated with a simple formula = 220—age. CI was defined as the inability to achieve the heart rate reserve (HRR). Pertaining to the therapy with beta-blockers: 80% of HRR was achieved in patients not taking beta-blockers and 62% in patients taking beta-blockers [6]. HRR was calculated as follows: HRR = [(Peak HR–HR at rest)/(220 –Age–HR at rest)] x 100% (Fig 2). We also assessed the number of patients who were able to achieve at least 70% of HRR, irrespective of beta-blocker therapy. The modified heart rate reserve (MHRR) value was determined with the following formula: MHRR = {Increment (%) from HR at rest to peak HR]/(220 –Age–HR at rest)} x 100 [7].

### 2.5. Statistical analysis

Continuous variables of the baseline characteristics were summarized with mean values +/- SD for normally distributed variables and median values and interquartile range (IQR) for non-normally distributed variables. Frequencies and percentages were used for categorical variables. Comparisons were made using the chi-squared test for nominal variables. Unpaired t-tests were used for comparison of normally distributed continuous variables and the Mann-Whitney U-test for non-normally distributed data.

The corresponding Kaplan–Meier curves for survival were also plotted and were compared using the log-rank test. Statistical significance for all analyses was determined as p<0.05. All analyses were carried out using the STATISTICA version 12 software.

## 3. Results

### 3.1. Characteristics of patients

The mean age of the analyzed population was 49 ± 12.4 years old (Table 1). The mean body mass index (BMI) of patients was 25.3 ± 4.1 kg/m2, however in patients with CI, mean BMI was significantly lower (24.7 ± 3.8 vs. 26.7 ± 4.4, p<0.005). Moreover, patients with CI had significantly lower resting HR in comparison to those without CI (81.5 ± 18.5 beats per minute; b.p.m. vs. 90.5 ± 16.9; p<0.005). The most frequent etiologies of ESLD were viral hepatitis B or C (45%) and alcoholic liver disease (18.1%). At the time of the cardiac consultation, the mean Child-Pugh score was 7.8 ± 2.1 units. 23.8% of patients achieved 15 or more points in the MELD score, however the percentage of patients with hepatocellular carcinoma (HCC), as a complication of ESLD, was relatively high (Table 1 and Fig 1). The most common complications of ESLD were gastroesophageal varices grade III-IV (31.1%) and ascites (26.9%). 62% of patients were treated with beta-blockers during the exercise stress test. We observed 18 post-liver-transplantation deaths (11.3%) in the study population within the mean follow-up period of over 2 years (736.5 ± 260.6 days). Patients with CI had more severe ESLD in regards to the Child-Pugh and MELD scores (Fig 1).

### 3.2. Exercise parameters

In the study group, the mean duration of exercise was 396 ± 137 seconds. The median achieved metabolic equivalents of tasks (METs) were 7.95 (6.97–10.2), and the mean percentage of patients who achieved ≥7METs was 76.8%. Diminished physical capacity, defined as the achievement of 4 METs or less, was diagnosed in 1.25% of patients. No significant differences in the length of the exercise stress test and physical capacity parameters between classes and groups were observed in regards to the Child-Pugh classification and MELD scores. However, statistically significant differences in those parameters were observed between different etiologies of liver disease. Patients with alcoholic cirrhosis had significantly shorter time of exercise (360 (259–408) vs 389 (297–482) vs 436 (343–540), respectively, p = 0.02) and achieved lower METs scores (7.0 (5.5–8.2) vs 7.6 (6.9–10.2) vs 8.7 (7.0–10.2), respectively, p = 0.01) in comparison to the viral hepatitis and other etiologies. Moreover, the smallest percentage of patients suffering from alcoholic cirrhosis achieved ≥ 7 METs in the exercise test when compared to viral hepatitis or other etiologies (58.6% vs 76.4% vs 86.4%, respectively, p = 0.02).Patients diagnosed with encephalopathy had a significantly shorter time of exercise (301 (221–398) vs. 410 (304–540); p = 0.002), achieved lower METs (7.0 (5.5–8.0) vs. 8.2 (7.0–10.2); p = 0.008) and fewer of them achieved ≥ 7 METs during the exercise test (61.3% vs. 80.3%; p = 0.03).

### 3.3. Chronotropic response

The chronotropic response of the analyzed population in regards to the severity and etiology of liver disease is presented in Tables 2–4. The results revealed that the parameters of chronotropic response during the exercise test depend on the progression and prognosis of ESLD. Patients classified to the Child-Pugh class A had a significantly higher maximal HR achieved during the exercise test in comparison to the Child-Pugh class B and C (Table 3). Moreover, the median achieved percentage of MPHR and percentage of patients who achieved ≥85% of the MPHR was the highest in the Child-Pugh class A when compared to the B and C (Table 3). HRR was the highest in the Child-Pugh class A in comparison to classes B and C (Table 3). The percentage of patients who met the criteria for CI was related to the Child-Pugh class and was the highest in class C (Table 3).

**Table 2:** Chronotropic response in the total analyzed population of patients with regards to the severity of liver disease correlating with the MELD score.

Chronotropic response	Total population (n = 160)	MELD<15 (n = 122)	MELD ≥ 15 (n = 38)	p-value
Rest HR, bpm (median; Q1-Q3)	81 (72–96)	82 (70–96)	81 (73–96)	0.92
Maximal HR, bpm (median; Q1-Q3)	140 (122–155)	141 (128–158)	130 (111–147)	0.02
Median achieved percentage of MPHR (median; Q1-Q3)	84% (72–89)	85% (76–90)	74% (65–86)	0.003
Achievement of ≥85% of MPHR (%)	49.4%	53.3%	36.8%	0.095
Chronotropic incompetence	68.8%	61.5%	92.1%	0.002
HRR (median; Q1-Q3)	65.8% (51.2–76.9)	67.7% (56.7–78.8)	51.2% (34.5–74.0)	<0.001
HRR<70%	59.4%	56.6%	68.4%	0.256
MHRR (median; Q1-Q3)	76.7% (60.8–91)	79.6% (65.8–93.3)	61.5% (46.5–75.8)	<0.001
MHRR<110%	92.5%	90.2%	100.0%	0.071

bpm–beats per minute, HR–heart rate, HRR–heart rate reserve, MHRR–modified heart rate reserve, MPHR—maximum predicted heart rate

**Table 3:** Chronotropic response in regards to the severity of liver disease correlating with the Child-Pugh classification.

Chronotropic response	Child-Pugh class A (n = 52)	Child-Pugh class B (n = 78)	Child-Pugh class C (n = 30)	p
Rest HR, bpm (median; Q1-Q3)	87 (74–98)	80 (70–95)	80 (69–96)	0.41
Maximal HR, bpm (median; Q1-Q3)	145 (135–159)	139 (120–153)	132 (111–146)	0.01
Median achieved percentage of MPHR (median; Q1-Q3)	87% (78–94)	81% (72–87)	79% (63–86)	<0.001
Achievement of ≥85% of MPHR	69.2%	41.0%	36.7%	0.002
Chronotropic incompetence	53.9%	74.4%	80.0%	0.02
HRR (median; Q1-Q3)	72.9% (59.8–85.7)	60.4% (50.5–72.1)	57.2% (34.4–75.5)	<0.001
HRR<70%	40.4%	69.3%	66.7%	0.003
MHRR (median; Q1-Q3)	84.8% (76.6–99.1)	72.6% (59.6–84.1)	72.4% (50.5–86.4)	<0.001
MHRR<110%	90.4%	93.6%	93.3%	0.86

bpm–beats per minute, HR–heart rate, HRR–heart rate reserve, MHRR–modified heart rate reserve, MPHR—maximum predicted heart rate

**Table 4:** Chronotropic response in regards to the etiology of liver disease.

Chronotropic response	Viral hepatitis (n = 72)	Alcoholic cirrhosis (n = 29)	Other etiology (n = 59)	p
Rest HR, bpm (median; Q1-Q3)	80 (70–90)	77 (70–89)	89 (73–107)	0.02
Maximal HR, bpm (median; Q1-Q3)	136 (117–150)	129 (116–143)	150 (135–165)	<0.001
Median achieved percentage of MPHR (median; Q1-Q3)	84% (71–91)	76% (68–84)	86% (78–89)	0.03
Achievement of ≥85% of MPHR	47.2%	24.1%	64.4%	0.002
Chronotropic incompetence	63.9%	82.8%	67.8%	0.18
HRR (median; Q1-Q3)	67.7% (48.4–81.6)	59.0% (42.6–66.7)	68.5% (59.0–76.2)	0.04
HRR<70%	54.2%	75.9%	57.6%	0.13
MHRR (median; Q1-Q3)	81.9% (61.7–96.6)	72.2% (59.6–80.5)	75.8% (59.5–92.6)	0.10
MHRR<110%	88.9%	100.0%	93.2%	0.16

bpm–beats per minute, HR–heart rate, HRR–heart rate reserve, MHRR–modified heart rate reserve, MPHR—maximum predicted heart rate

Considerable differences in chronotropic response, depending on the MELD score, could also be observed (Table 2). Patients with MELD score ≥ 15 had a significantly lower maximal HR, lower median achieved percentage of MPHR, lower HRR and lower MHRR. Moreover, patients with MELD score ≥ 15 met the criteria for CI more frequently (Table 2).

Chronotropic response differed significantly according to the etiology of ESLD (Table 4). In comparison to the viral hepatitis and other etiologies of ESLD, patients with alcoholic cirrhosis had a significantly lower rest HR, lower maximal HR, lower median achieved percentage of MPHR, lower percentage of patients who achieved ≥ 85% of MPHR and lower HRR (Table 4).

Furthermore, we noticed obvious consequences of beta-blocker usage, i.e. significantly lower rest HR, lower maximal HR, lower median achieved percentage of MPHR, lower percentage of patients who achieved ≥ 85% of MPHR and lower HRR. Moreover, chronotropic parameters differed significantly in regard to presence of CI (Table 5). Patient with CI had significantly lower values of rest HR, maximal HR, median achieved percentage of MPHR, lower percentage of patients who achieved ≥ 85% of MPHR, lower HRR, MHRR and higher percentage of patients with HRR<70% and MHRR<110% (Table 5).

**Table 5:** Chronotropic response in regards to presence of chronotropic incompetence.

Chronotropic response	CI+ (n = 110)	CI- (n = 50)	p
Rest HR, bpm (median; Q1-Q3)	78 (69–91)	88 (76–103)	0.003
Maximal HR, bpm (median; Q1-Q3)	132 (114–145)	155 (144–168)	<0.0001
Median achieved percentage of MPHR (median; Q1-Q3)	77% (68–86)	93% (86–98)	<0.0001
Achievement of ≥85% of MPHR	31.8%	88%	<0.0001
HRR (median; Q1-Q3)	57.5% (41.2–67.2)	83.4% (73.5–92.5)	<0.0001
HRR<70%	78.2%	18%	<0.0001
MHRR (median; Q1-Q3)	68.2% (53.2–81.8)	93.6% (82–107.9)	<0.001
MHRR<110%	97.3%	2.7%	0.002

bpm–beats per minute, HR–heart rate, HRR–heart rate reserve, MHRR–modified heart rate reserve, MPHR—maximum predicted heart rate

### 3.4. CI and prognosis of OLT recipients

**Fig 3:**
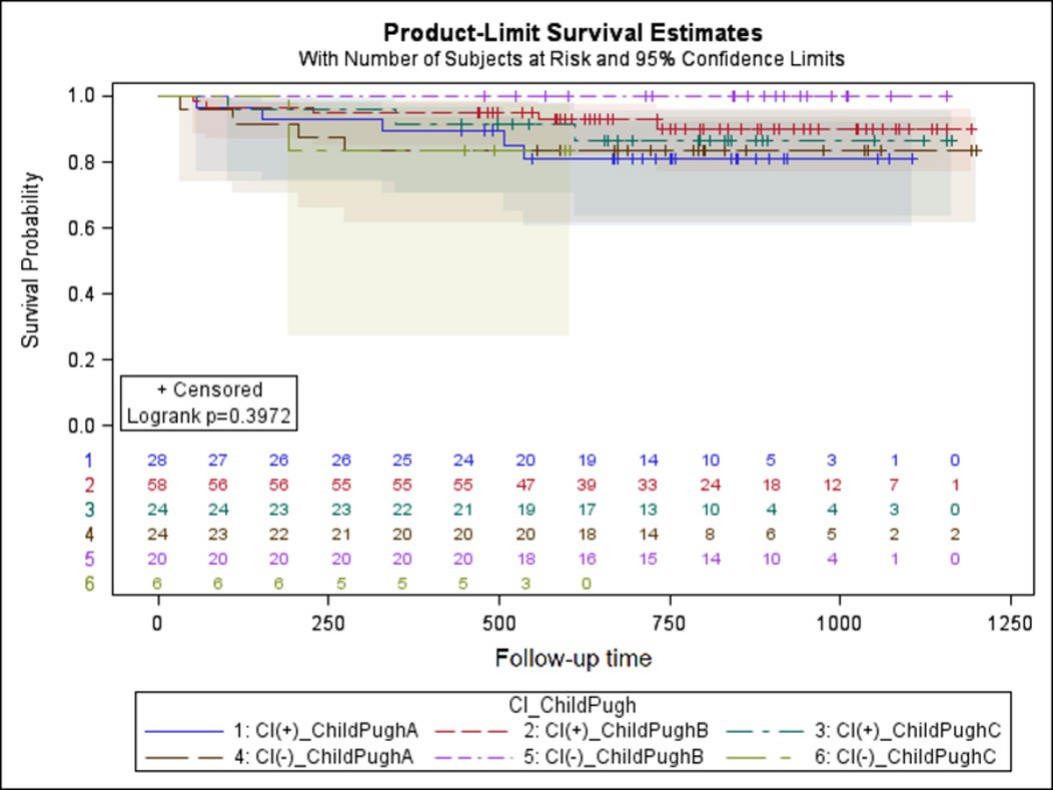
Kaplan-Meier curves for survival of OLT recipients regarding to class of Child-Pugh scale.

**Fig 4:**
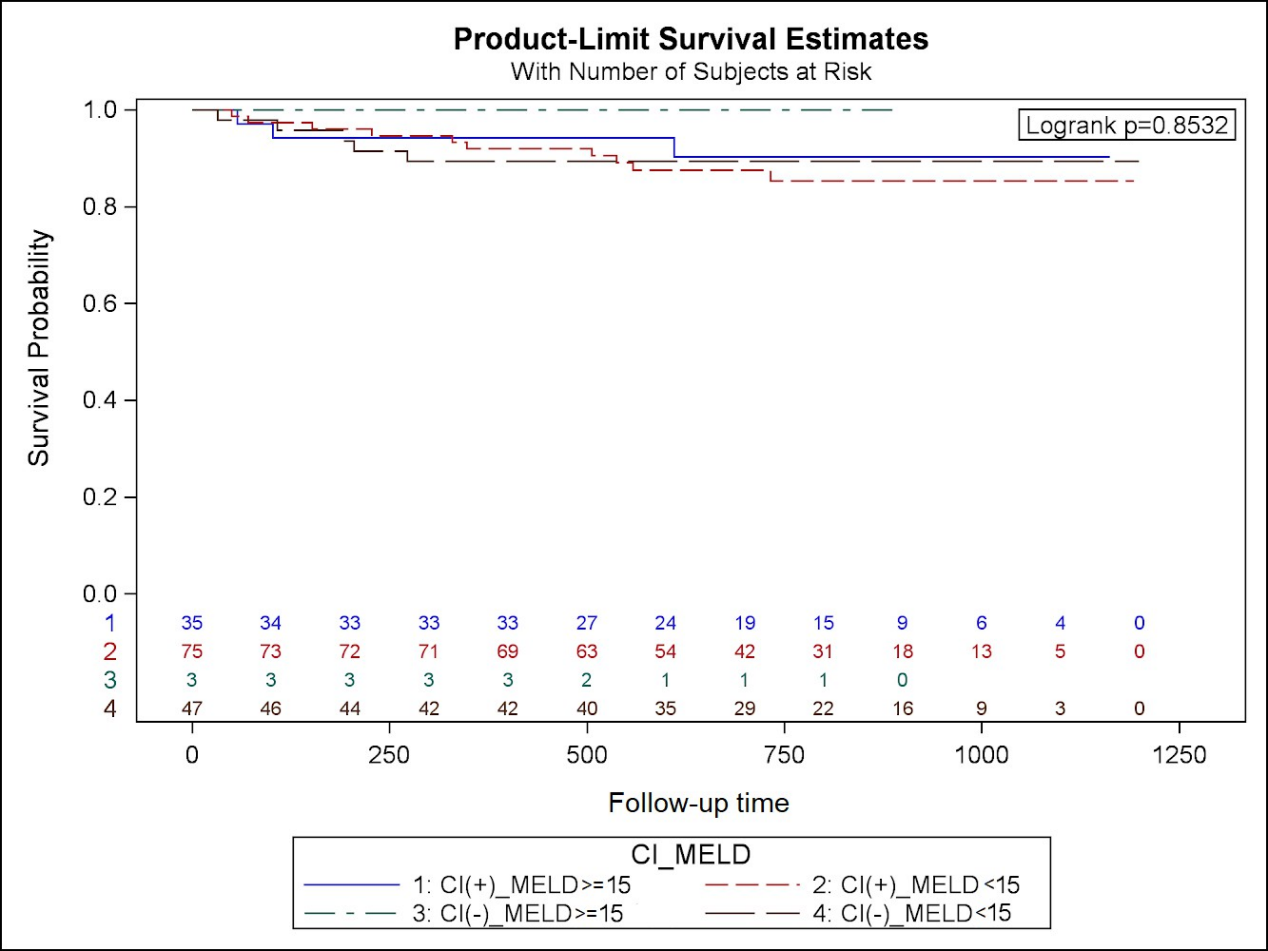
Kaplan-Meier curves for survival of OLT recipients regarding to MELD score.

**Fig 5:**
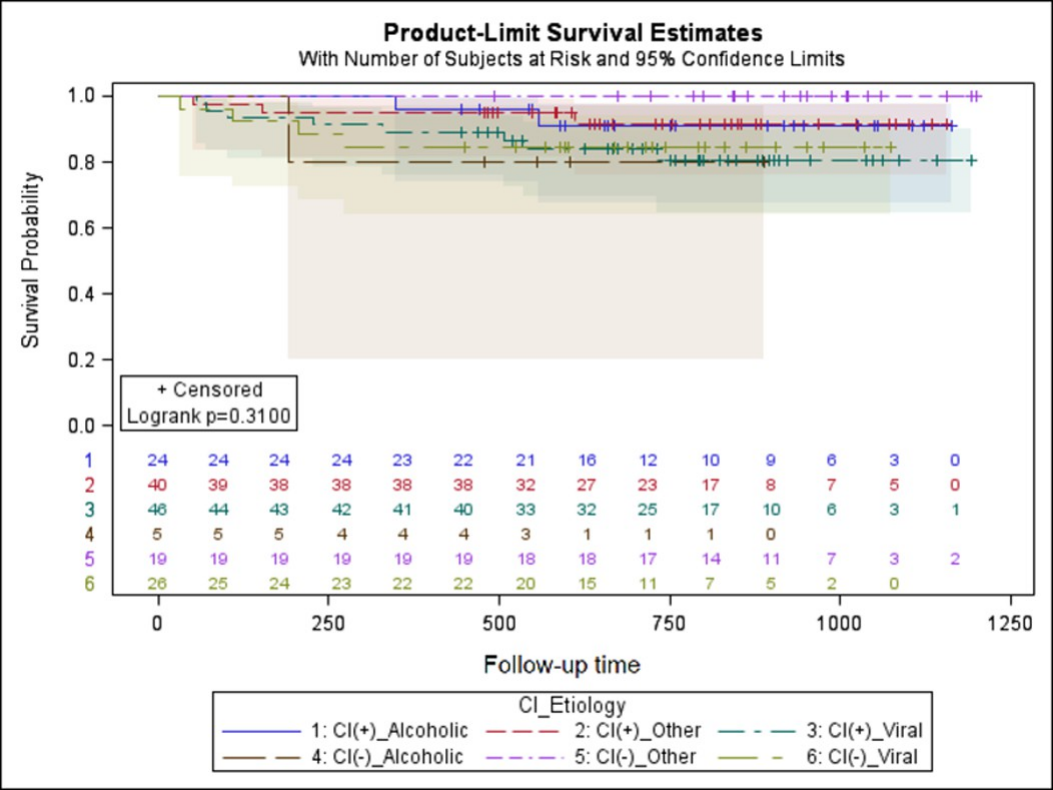
Kaplan-Meier curves for survival of OLT recipients regarding to etiology of ESLD.

The Kaplan-Meier curves for survival of OLT recipients are plotted regarding to presence of CI and class of Child-Pugh scale (Fig 3), MELD score (Fig 4) and etiology of ESLD (Fig 5), however no significant relation was found.

## 4. Discussion

In this study we report a relatively high prevalence of CI among liver transplant candidates, however it did not have a significant impact of their long-term survival post OLT [5, 7]. The current analysis showed a significantly altered chronotropic response in regard to the severity of liver disease according to both Child-Pugh and MELD scores. This observation confirmed the thesis that CCM is a late onset complication of liver cirrhosis, and ESLD patients are at risk of its development.

Several studies proposed the dobutamine stress echocardiographic examination (DSE) as a reliable method for the cardiovascular assessment of liver transplant candidates, which also enables the diagnosis of CI [5, 7]. However, the exercise stress test is a more familiar method for cardiologists and is available in more centers worldwide. It should be emphasized, that DSE in ESLD patients with ascites is of limited use due to a poor acoustic window. Singhal et al. performed DSE in 111 liver transplant candidates and found 48.75% patients with CI. CI was defined as the inability to reach 85% of MPHR for age and sex at the maximum dosage of dobutamine (40 /kg/min) in the study of a DSE prior to the OLT [5]. Patients with CI in this study were older and had less advanced ESLD (mean MELD score 14.3 points) when compared to the recent results. Rudzinski et al. performed DSE in 284 ESLD patients and reported, that 23% of patients were not able to achieve the target HR, despite the maximum doses of dobutamine and atropine and HRR<70% was observed in 24% of patients, 56% of which were on beta-blocker therapy [7]. In comparison to the current study, the population analyzed by Rudzinski et al. was older, had more advanced ESLD (the mean MELD score was 21.1 ± 7.5) and a smaller percentage of patients received beta-blockers (40%) [7]. The results of our study reinforce the previous suggestion, that MHRR, as a new index independent of beta-blocker effects, may be useful in the determination of the target HR not only for DSE, but also for cardiac exercise stress testing and the diagnosis of CI [7]. The role of beta-blockers in ESLD patients is currently extensively studied, after the initial reports of increased risk of death after variceal bleeding. However, recent studies confirmed their beneficial effect on survival in patients with cirrhosis and ascites (including those with refractory ascites) [8], in liver transplant candidates [9] as well as in patients with acute-on-chronic liver failure [10]. Thus, the number of ESLD patients treated with beta-blockers will be increasing.

The pathophysiologic mechanisms of CI are associated with autonomic neuropathy [3, 11]. In the context of liver transplant candidates, it has to be emphasized, that the anaesthesia itself has a significant influence on perioperative autonomic function, which may lead to haemodynamic instability during surgery in patients with an autonomic neuropathy [12]. Moreover, it has been proven, that end-stage liver cirrhosis patients with a definite autonomic dysfunction have a worse hyperkinetic circulation (increased cardiac output with decreased systemic vascular resistance) and a higher prevalence of the post-reperfusion syndrome (PRS) during liver transplantation. Furthermore, patients with autonomic neuropathy have arterial hypotension at the time of the reperfusion period more frequently and have a higher requirement for norepinephrine and inotropic support during OLT [11]. Currently, there are no explicit guidelines for the perioperative management of patients with CI. It has been shown that the use of specific anaesthetics is not related to the perioperative hemodynamic depression and may be useful in patients with a significant risk of intraoperative cardiovascular adverse events. Moreover, when the elimination of the analgesic effect in the recovery period is faster, the increased activation of the sympathetic nervous system may reduce the requirement for vasopressors in order to support the hemodynamic parameters [12, 13]. It has been reported, that in diabetic patients the pre-operative assessment of resting heart rate variability correlates with the hemodynamic stability at the time of anaesthesia induction. Therefore, the pre-operative assessment of the cardiac autonomic function, for instance, using the cardiac exercise stress test, may enable the development of tailor-made anaesthesia for patients with ESLD and autonomic dysfunction and improve the safety of OLT.

## 5. Conclusions

The prevalence of CI among liver transplant candidates seems to be higher than previously reported, but does not seem to influence the long-term survival after OLT. The altered chronotropic response may differ in regard to the severity of liver disease depending on both Child-Pugh and MELD scores. The exercise stress test is a reliable, safe and useful tool for the diagnosis of CCM in liver transplant candidates and should be included in the standard cardiovascular assessment prior to OLT. Diagnosis of CCM in patients qualified for liver transplantation will allow individual adaptations of the perioperative management and implementation of effective post-reperfusion prophylaxis and management of intraoperative hemodynamic disturbances by selecting appropriate pharmacological agents at adjusted doses, which will also prevent the excessive increase in blood pressure.

## 6. Limitations of the study

Our study does not include information about the prevalence of PRS and in-hospital mortality of patients, due to the fact that there is no clear definition of PRS in the current literature, especially concerning the use of catecholamines prior to reperfusion. We are not able to confirm the exact percentage of patients with CCM, due to an unclear and unestablished definition. Moreover, data from the Cardiopulmonary Exercise Testing (CPET) and detailed analysis of the usage beta-blockers in that population of patients would add additional value to the study and should be concerned in further research on that topic.

## List of abbreviations

CCM: Cirrhotic cardiomyopathy
CI: Chronotropic incompetence
DSE: dobutamine stress echocardiographic examination
ESLD: End-stage liver disease
HCC: Hepatocellular carcinoma
HR: Heart rate
HRR: Heart rate reserve
MELD: Model for End-Stage Liver Disease
METs: Median achieved metabolic equivalents of tasks
MHRR: Modified heart rate reserve
MPHR: Maximal predicted heart rate
OLT: Orthotopic liver transplantation
PRS: Post-reperfusion syndrome
RAAS: Renin-angiotensin-aldosterone system
